# Taking action on developmental toxicity: Scientists’ duties to protect children

**DOI:** 10.1186/1476-069X-11-61

**Published:** 2012-09-10

**Authors:** Kristin Shrader-Frechette

**Affiliations:** 1 Department of Biological Sciences, University of Notre Dame, Notre Dame, IN, 46556, USA; 2 Department of Philosophy, 100 Malloy Hall, University of Notre Dame, Notre Dame, IN, 46556, USA

**Keywords:** Developmental toxicity, Ethics, Justice, Policy, Risk, Scientists’ duties

## Abstract

**Background:**

Although adaptation and proper biological functioning require developmental programming, pollutant interference can cause developmental toxicity or DT.

**Objectives:**

This commentary assesses whether it is ethical for citizens/physicians/scientists to allow avoidable DT.

**Methods:**

Using conceptual, economic, ethical, and logical analysis, the commentary assesses what major ethical theories and objectors would say regarding the defensibility of allowing avoidable DT.

**Results:**

The commentary argues that (1) none of the four major ethical theories (based, respectively, on virtue, natural law, utility, or equity) can consistently defend avoidable DT because it unjustifiably harms, respectively, individual human flourishing, human life, the greatest good, and equality. (2) Justice also requires leaving “as much and as good” biological resources for all, including future generations possibly harmed if epigenetic change is heritable. (3) Scientists/physicians have greater justice-based duties, than ordinary/average citizens, to help stop DT because they help cause it and have greater professional abilities/opportunities to help stop it. (4) Scientists/physicians likewise have greater justice-based duties, than ordinary/average citizens, to help stop DT because they benefit more from it, given their relatively greater education/consumption/income. The paper shows that major objections to (3)-(4) fail on logical, ethical, or scientific grounds, then closes with practical suggestions for implementing its proposals.

**Conclusions:**

Because allowing avoidable DT is ethically indefensible, citizens---and especially physicians/scientists---have justice-based duties to help stop DT.

## Background

In Spring 2012, organizers of PPTOX III---a conference focused on integrating "the role of environmental exposures and nutrients during development on subsequent diseases/dysfunctions later in life"--- issued a white paper. So far, nearly 100 physicians/scientists throughout the world have co-signed it. Reviewing classic scientific research on how developmental exposures to environmental chemicals can cause later disease/dysfunction, the white paper draws two main conclusions. One is that children’s in-utero and early-postnatal-developmental periods are “particularly sensitive to developmental disruption by nutritional factors or environmental-chemical exposures, with potentially adverse consequences for health later in life.” A second conclusion is that children’s heightened developmental sensitivity requires a new “policy and public health response” of “both research and disease-prevention strategies” [1].

### Developmental toxicity (DT)

While the white paper provides the scientific basis for special child-developmental protection against environmental chemicals, this paper provides a preliminary ethical basis for protection. It argues that, to varying degrees and for slightly different reasons, citizens, physicians, and scientists each have justice-based, sometimes overlapping, duties to help stop developmental toxicity or DT.

DT refers to the fact that “many of the major diseases---and dysfunctions---that have increased substantially in prevalence over the last 40 years seem to be related in part to developmental factors associated with…exposures to environmental chemicals” [1]. Scientists confirm that, despite the need for much more research to reduce various scientific uncertainties, the concept of the “developmental origins of [much] health and disease…is sufficiently robust and repeatable across species, including humans” [1], that toxin-induced “epigenetic modifications can be passed from one cell generation to the next and, in some cases, when germ cells are targeted, can be transgenerationally transmitted” [2]. These adverse effects, however, “may not be apparent during a latent period which may last from months to years or decades,” and they can result from “a combination of developmental stressors…[that] cause effects jointly with similar or other exposures.” These harms---manifested as “increased incidence,…earlier onset, or an increased severity” of disease or dysfunction---include “obesity, diabetes, hypertension, cardiovascular disease, asthma and allergy, immune and autoimmune diseases, neurodevelopmental and neurodegenerative diseases, precocious puberty, infertility, some cancer types, osteoporosis, depression, schizophrenia, and sarcopenia” [1].

Although all environmental exposures likely causing DT have not been fully confirmed, low-dose environmental chemicals known to cause DT include “especially endocrine disrupting chemicals” or EDCs. “Examples of EDCs known to alter disease susceptibility as a result of developmental exposures in animal models include bisphenol A (from polycarbonate plastics), phthalates (a softener in plastics), some organophosphate and organochlorine pesticides, nicotine (tobacco smoking), air pollution, perfluorooctane compounds (stain and water repellents), and polybrominated diphenyl ethers (flame retardants) – all chemicals that are found in detectable concentrations in blood or urine samples from most people” [1].

### Using a thought experiment to help decide how to respond to DT

The preceding list of developmental toxins, however, is not complete. Also, because much DT testing remains to be done, some scientists are uncertain about DT and its potential epigenetic effects. How should scientists/society respond, given such unknowns?

When it is impossible or impractical for scientists to rapidly conduct real-world experiments on some question, they often use thought experiments---apriori, rather than empirical, assessments using only reason and imagination. Richard Feynman famously described thought experiments as more elegant than physical ones. Galileo’s “tower” thought experiment showed that, contrary to Aristotle, objects of different masses fall at the same acceleration. Maxwell’s “demon” showed that, contrary to the second law of thermodynamics, entropy could be decreased. Einstein used Schrödinger's “cat” to show that, contrary to Copenhagen interpretations of quantum mechanics, observation does not break quantum-state superposition.

Ethicists also use thought experiments. Judith Thomson’s “transplant surgeon” showed that, contrary to some utilitarians, one cannot deliberately kill an innocent person to save more lives. Philippa Foot’s “trolley” showed that, contrary to some egalitarians, one could allow one death in order to save more people, provided the victim was not used as a means to this end.

Would a thought experiment also help clarify how scientists/society ought to respond to DT---to environmental-chemical exposures that may put children at risk of later disease and dysfunction? To answer this question, consider a thought experiment about another potential harm to children.

Suppose you are on vacation in Brazil, taking an early-morning walk. Crying, a tiny boy’s well-dressed “aunt” approaches you, saying she is late for work. She apologizes for bothering you, is clearly distraught, and says she fears losing her job because of her lateness, unless someone takes her visiting “nephew” to his mother. The “aunt” pleads for help, gives you the child and his “address,” then offers you money for taking him “home.” Should you help the “aunt,” take the child to the address, but refuse the money? Or should you take the child to the police?

On one hand, the Brazilian “aunt” may be telling the truth and may need a Good Samaritan. On the other hand, the child may be at risk of organ trafficking. Given the shortage of available organs for transplant, and the millions of “street children” in nations like Brazil, the World Health Organization estimates that organ trafficking accounts for up to 10 percent of all organ transplants. Part of a trillion-dollar annual global-economic output in illicit trade, organ trafficking annually causes thousands of murders among many of those whose organs are harvested for resale [3,4].

What you ought to do about the Brazilian child may illuminate what scientists/society ought to do about DT. After all, although organ transplants and industrial/agricultural chemicals serve important goods, both pose at least 7 serious---and similar--risks. Both involve potential harm, uncertainty, innocent victims, lack of child consent, and high stakes---only “one chance” to possibly avoid organ-harvester murderers or to “develop a brain” [5]. Both also involve questions such as whether to minimize false-negative risks (to children) or false-positive risks (to the aunt/chemical-industry), and whether to make health-economics tradeoffs.

Most informed people probably would find it easier to decide what to do in the Brazilian-child case than in the DT situation, partly because of organ-trafficking publicity. They likely would not give an innocent child to possible traffickers. Yet this commentary argues that---according to all major ethical theories---the answer regarding whether citizens/physicians/scientists should allow avoidable DT is almost as clear as whether to deliver the Brazilian child to his “home.” In both cases, justice and the vulnerability of children argue for their protection.

### Scientific uncertainty about organ trafficking and DT

Why does it seem more difficult to decide the DT, than the Brazilian-child question? One reason, already noted, is that the relevant science is still unfolding. A second reason is that organ trafficking involves threats to specific individuals, while scientists typically can document subtle DT harm mainly to populations, although individuals are affected. Third, human studies reveal organ trafficking, whereas mainly animal studies reveal DT. Fourth, organ trafficking is more obvious to laypeople than is DT. Fifth, because of this obviousness, citizens might claim that in the organ-trafficking case, it is better to risk false positives (false assertions of child harm) than false negatives, whereas in the DT case, *scientists* might claim it is better to risk false negatives (false assertions of no DT harm to children), than false positives, because scientists typically have more aversion to false positives [6,7]. Sixth, although organ traffickers obviously have no rights to economic gain from their activities, industries (that seriously harm no one) do have such rights. If these industries are unjustly accused, they could face economic harm from DT regulation, but if possible DT victims are not protected, innocent, non-consenting children (and future generations, if epigenetic change is heritable) could face health harm---a possible stealth DT pandemic. Yet, given global recession and government-research-funding cutbacks, arguments for increasing DT funding, so as to reduce all these DT-associated uncertainties, face an uphill battle. The result?

Needed DT-related science is not getting done, and many uncertainties about DT remain. This is partly because of the “Matthew Effect” [8], that most scientific studies---of substances on the US Environmental Protection Agency’s (EPA’s) risk-based-priority ranking of high-production-volume (HPV) chemicals---focus on the top 10–20 chemicals. Already-overstudied chemicals are studied more, whereas risky chemicals, ranked 50^th^ or 90^th^ among roughly 80,000, are rarely studied [8]. A second cause of DT uncertainty is that only 7 percent of HPV chemicals have ever been assessed for developmental effects or toxicity to children [9]. Third, under the innocent-until-proved-guilty assumption, at least 1000 new chemicals enter the market annually, giving scientists little time to study them before they may cause serious harm [10].

A fourth reason for uncertainty about DT is scientists’ disagreement over causal inferences. British statistician Austin Bradford Hill [11] introduced 9 causal-inference guidelines: strength of the mathematical association, biological plausibility (underlying mechanisms), coherence, consistency with other results, specificity of results (one-to-one relationships), temporality (causes’ preceding effects), biological gradients (dose–response curves), experimental evidence, and analogies with causal precedents.

So-called “black-box epidemiologists” (the majority camp) emphasize Hill’s first guideline and use mathematical/statistical measures like relative risk to assess cause-effect relationships, whereas so-called “eco-epidemiologists” (the minority camp) are methodological pluralists who emphasize the second guideline and often seek cause-effect mechanisms. Although black-box epidemiologists emphasize statistical evidence, even for purely-observational data, are more eager to avoid false positives, and focus on disease treatment---eco-epidemiologists avoid using only statistical tests of observational data, are more eager to false negatives, and focus more on disease prevention. Unsurprisingly, these camps often disagree about harm from specific chemical exposures [12].

### First of four arguments: Dominant ethical theories disallow DT

Despite some disananogies between organ trafficking and DT exposure, and despite some scientific uncertainties about DT, the ethics is reasonably clear. Any one of at least four different ethical arguments, given in subsequent pages, is alone sufficient to show that it is prima-facie unethical to allow avoidable DT. (To call something prima-facie unethical means (i) that a general ethical principle affirms it is unethical; (ii) that the burden of proof is on anyone wishing to justify a case-specific override of this prima-facie principle; and (iii) that anyone who disagrees (with a case-specific application of the principle) must provide precise, compelling, ultima-facie (all-things-considered) arguments to the contrary [13,14]. Such ultima-facie arguments might be, for instance, that allowing DT, given a specific chemical compound/case/ time/set of circumstances, would cause less human death/disease/dysfunction than not allowing it).

Why do the four arguments (given in subsequent pages) establish merely prima-facie principles against allowing DT? Because ethical principles must be applicable to a wide variety of situations, by definition they do not take into account the millions of case-specific or conflict-specific factual and moral considerations---ultima-facie considerations---that might override them and cause exceptions. Instead, moral principles establish merely prima-facie claims that put the burden of proof on violators.

The four different ethical arguments, each sufficient to show that allowing avoidable DT is prima-facie unethical---focus, respectively, on (1) ethical theory, (2) just political theory, (3) DT causality, and (4) DT benefits. (1) The ethical-theory argument has four parts and argues that no major ethics codes (Aristotelian, Thomistic, utilitarian, egalitarian) allow avoidable DT because, even if epigenetic change is not heritable, DT unjustifiably harms individual human flourishing, life, equality, and good. (2) The just-political-theory argument is that no major political codes allow unequal “takings” from the biological commons, as would occur if DT-induced epigenetic change is heritable. (3) The DT-causality argument is that---even if epigenetic change is not heritable---all citizens, and scientists/physicians in particular, have justice-based duties to take action on avoidable DT because they helped cause it. (4) The DT-benefits argument is that---even if epigenetic change is not heritable---all citizens, and scientists/physicians in particular, have justice-based duties to take action on avoidable DT because they benefit from it. After presenting these four basic arguments, the paper shows that all major objections to them are logically/ethically/scientifically flawed.

To assess argument (1) above, consider four main ethical codes---classical Aristotelian virtue theory, medieval Thomistic natural-law theory, modern/contemporary Millian utilitarianism, and modern/contemporary Rawlsian egalitarianism. A very quick, simple survey reveals that all these ethical theories would mandate that allowing avoidable DT is prima-facie indefensible.

### Aristotelian ethics

For Aristotelians, the purpose, meaning, goal, or telos of life is human flourishing or eudaimonia, the desired end of all human actions. For Aristotelians, humans achieve this end or telos of flourishing by having a virtuous character. And they attain a virtuous character through practicing the virtues---such as justice and courage. Because DT threatens the fundamental telos or end of human flourishing or eudaimonia---given its increasing disease susceptibility, adverse neurological/other effects, epigenomic disruption, and possible transgenerational effects [2,15-18]---consistent Aristotelians would not allow DT. Moreover, in the Aristotelian world, where the virtue of courage is a key means to flourishing or eudaimonia, those who lack the courage to protect others, such as innocent children, will themselves not attain eudaimonia. Therefore those lacking courage behave unethically, apart from the indefensible harm they allow to children.

Suppose someone objected that allowing DT-inducing environmental toxins promoted overall economic welfare, therefore human flourishing? Consistent Aristotelians would reject this objection because they believe money is merely a medium of exchange, something able to corrupt people and natural exchanges, hence something unable to capture fundamentally incommensurable and superior values---like human flourishing. Aristotelians believe the fundamental ethical end, telos, or eudaimonia is not captured through cost-benefit calculations. Therefore, to show that allowing DT is ethical, for Aristotelians, would require showing that DT promoted greater virtue and eudaimonia or flourishing. Yet there is no reason to believe that allowing DT would lead to greater virtue and flourishing*,* given the harms it causes [19].

### Natural-law ethics and alleged “free-market” objections

A second major class of ethical theorists, consistent followers of Thomas Aquinas’ natural-law ethics, likewise must reject avoidable DT. For them, universal law---written on human hearts, discoverable by human reason---binds people/government to act in accord with it, especially its fundamental tenet to preserve human life and authentic happiness [20]. Because DT threatens life and happiness---just as it threatens Aristotelian flourishing---natural-law theorists cannot allow avoidable DT.

Of course, anti-regulatory political theorists---like attorney Cass Sunstein, current (2012) administrator of the US Office of Information and Regulatory Affairs, whose research has been funded by right-wing think tanks, like the American Enterprise Institute---reject this ethics. He and other alleged “free-market” environmentalists would likely claim that allowing DT contributes to preserving human life. Sunstein’s argues that (1) monies spent on regulations “produce less employment and more poverty,” (2) “wealth buys longevity,” and therefore health-related regulations cost money, “increase risk,” thus kill people [21].

However, Sunstein’s alleged-free-market objection to stopping DT in invalid because it commits three logical fallacies of false cause in premises (1–2) above. The first fallacy consists of assuming in premise (1) that regulations reduce employment. Yet health-related regulations are neither necessary nor sufficient conditions for reduced overall employment. Instead, health-related regulations typically increase overall employment or shift it from one sector/industry to another, with no net job loss. For instance, workers often move from old/dirty to new/clean technologies, with no overall job loss, partly because many clean technologies such as solar/wind are more labor intensive, per kilowatt of electricity produced. Cleaner technologies also often save lives and therefore jobs [22,23].

A second false-cause fallacy in Sunstein’s premise (1) is the assumption that industrial profits are always spent to increase employment, an assumption falsified by the last half-century of US economic history. A third false-cause fallacy occurs in premise (2), that “wealth buys longevity.” On the contrary, research shows mortality is very strongly associated with societal income-inequality, not with either per-capita or median income [24].

These three false-cause fallacies arise partly because alleged “free-market” environmentalists ignore relevant facts/norms, such as that higher employment has little value for people increasingly made ill by DT, or that higher employment does not excuse injustice to innocent children. It did, one could justify employing many people as “pushers” to children. This supposed free-market argument likewise is invalid for a fourth reason: It begs the question that cost-benefit analysis is the sole test for regulations. A simple counter example shows it is not: Law requires expensive trials and possible prosecution/incarceration/death for accused murderers. Yet, criminologists agree these requirements are rarely cost-effective because most murders are not serial offenders, hence pose no future threat to society. Instead, society tries them because justice requires it. If so, justice trumps cost-effectiveness and therefore alleged “free-market” environmentalism.

Apart from its four logical fallacies, the alleged “free market” objection to avoiding/preventing DT also fails scientifically. Why? Adam Smith and virtually all economists and ethicists agree that economically-efficient (and ethical) market transactions require full information and fully voluntary exchanges. Without them, Coase’s famous theorem is inapplicable. (Coase’s theorem---a key basis for economic analysis of government regulation---is that, regardless of how property rights are initially apportioned, if there are no transaction costs, and if people can trade in an externality, then bargaining will lead to an economically-efficient outcome.) Yet obviously consumer-market behavior regarding pollutants is neither fully informed nor fully voluntary, partly because polluters often mislead about harm, fight labeling and right-to-know requirements, and resist government/consumer information-gathering [25-28]. If so, supposed “free-market” proponents apply their views to precisely the cases (DT cases without fully informed, voluntary exchanges) that, economists agree, generate no economically-efficient outcomes. Indeed, poor or poorly educated consumers often have neither equal bargaining power nor ability to correct disinformation and skewed market forces. If not, misapplied free-market objections really offer a wolf’s argument in sheep’s clothing. The sheep is market reasoning; the wolf is “might makes right,” defending conclusions that allow harm to poor/innocent people on false grounds that harming them increases overall wealth/employment. Given alleged “free-market” environmentalism’s scientific/ethical flaws, it is unsurprising that consistent free-market economists reject claims that regulations kill people [29]. Thus, supposed “free-market” objections to avoiding/preventing DT are invalid. They fail on logical, scientific, and ethical grounds.

### Utilitarian ethics

A third major group of ethicists, modern/contemporary utilitarians, maintains ethical actions are those that achieve the greatest good for the greatest number of people, as measured by preferences. They likewise cannot consistently defend allowing avoidable DT because DT death/disease, and people’s uncertainty/anxiety regarding whether their loved ones will be DT victims, both harm the greater good. Why? Harms to minorities, like children, hurt the majority. This why utilitarian John Stuart Mill [30] rejected “the tyranny of the majority.”

However, an objector might claim that societal inequities from DT do not reduce overall welfare because health-related regulations cause unemployment, thus kill people. Yet, as the previous criticisms of objections to natural-law arguments for avoiding/preventing DT reveal, the alleged “free-market” objection fails to justify allowing avoidable DT because its premises are factually false, and its inferences are invalid.

### Egalitarian ethics

A fourth main group of ethicists, modern/contemporary egalitarians, likewise would reject avoidable DT. Egalitarians, like the late Harvard ethicist John Rawls [31], say that because equal opportunity and liberty are primary ethical goals, unavoidable societal inequalities should be arranged so as to benefit the least-well-off, the most vulnerable. Consequently they would reject avoidable DT because it thwarts liberty and equal opportunity, especially among least-well-off victims, like children.

Nevertheless, objectors might counter egalitarianism by appeal to the alleged “free-market” objection, already considered/rejected. Yet the free-market objection relies on rejecting equal opportunity for the sake of alleged economic benefits. Rawls, however, argues no one would sanction equal-opportunity violations unless she were certain not to be a victim, a fact demonstrating the self-interested bias---and invalid arguments---of those who reject egalitarian ethics.

As the preceding considerations reveal, all main ethical theories (based on virtue, equality, overall preferences, and equality) reject allowing avoidable DT, but on different grounds and with different degrees of stringency. Aristotelian virtue ethicists, Thomistic natural-law theorists, and Rawlsian egalitarians would all reject avoidable DT because of its harms to basic goods, respectively, flourishing, life, and equality. Millian utilitarians are a bit less stringent (in disallowing avoidable DT) than are ethicists in the other three groups, because utilitarians in principle allow money/harm tradeoffs. However, even utilitarians would find it nearly impossible to show that most people’s preferences would trade DT harm to their children for money.

### Second argument: Major political theories disallow DT

Besides all four major ethical theories, dominant political theories---if they are consistent---must disallow avoidable DT. Whether socialist, Marxist, libertarian, capitalist, or anarchist, virtually all political theorists accept John Locke’s account of property rights and his labor theory of value. That is, they all maintain human labor/merit/effort creates property rights in things, and that one cannot have full property rights over anything one’s labor has not created---although different groups may disagree over what constitutes labor/merit/effort, and how far property rights extend. Because human labor did not create common natural/biological resources (such as land and genes), full property right to them are impossible and, instead, Lockeans argue one can use natural/biological resources only under the constraint of an equal-opportunity criterion---provided “as much and as good” is left for all other people, including future generations. While political theorists may disagree over precisely what is “as much and as good” for future people, they all agree with this Lockean account of property rights and its equal-opportunity criterion for natural/biological resources [32]. Otherwise, despots could illegitimately claim full rights to resources needed for others’ survival. Because avoidable DT harms resources (like genomic stability)---possibly for future generations, if epigenetic changes are heritable---avoidable DT harms are never permissible. One never has rights to “take” that over which (like biological resources) one has no property rights.

But doesn’t society recognize civil property rights/patents over natural resources like land and genes? While social/civil convention accepts such legal rights as a matter of convenience, even conservative ethical/legal theorists (including William Blackstone) agree that no rational grounds (only grounds of “might makes right”) exist for claiming property rights over things not created by labor. Instead, they admit property “rights” to biological resources often originated from fraud, violence, theft, bullying, or colonialism---then were “transferred” through the ages. Thus, virtually all “transfers” have been problematic because original “owners” often had the equivalent of stolen goods, violating Locke’s as-much-and-as-good proviso. If so, many current conventional property rights (to biological-commons resources) are as ethically indefensible as conventions of racism, sexism, or homophobia. But if property rights to natural biological resources---such as genomic stability/health---are impossible, and if those who threaten genetic integrity (because of the heritability of epigenetic change) therefore have no rights to do so, then avoidable DT is ethically impermissible [33], and citizens/scientists should not allow it.

### Third argument: Helping cause DT creates greater DT duties

Apart from ethical theory, justice-based reasons also show that citizens, and especially physicians/scientists, should not allow avoidable DT. The DT-causality argument is that---even if epigenetic change is not heritable---all citizens, and scientists/physicians in particular, have justice-based duties to take action on avoidable DT because they helped cause this harm to children. How so? Virtually everyone contributes to air pollution, and virtually everyone uses/purchases products containing bisphenol A, phthalates, organophosphate and organochlorine pesticides, nicotine, perfluorooctane compounds, and polybrominated diphenyl ethers–-all chemicals known to cause DT [1]. Because everyone contributes to DT through pollution and product use, everyone has duties to help stop this harm they cause. The ethics is basic: if you broke it, you should fix it.

Moreover, because democracy is not a spectator sport, and because all citizens in a democracy are responsible for being politically active, so as to help stop public harms, all citizens have duties to help stop avoidable DT. Those who have never even attempted to help stop avoidable DT have special duties to do so, precisely because they bear a greater responsibility for helping cause DT because of their inaction and their failure to exercise the duties of citizenship. Thus, although everyone who helps cause DT has prima facie duties to help stop it, those duties differ, ultima facie and individual to individual, as a function of factors such as one’s fractional contribution to DT, one’s knowledge, profession, time, expertise, etc. [34].

### Scientists’/physicians’ greater abilities generate greater duties

Although scientists/physicians share duties with other citizens to help stop DT because of helping cause it, they have special duties, all other things being equal, because they have greater ability/expertise/intellectual-professional resources (than laypeople) to help stop DT. As all professional ethics codes note, all other things being equal, greater ability to prevent some harm generates greater responsibilities to do so [35]. The American Medical Association, for instance, says that because of their “skills and competency,” physicians should “educate the public and policy about present and future threats to the health of humanity” and “advocate for social, economic, educational, and political changes that ameliorate suffering and contribute to human well-being” [36].

### Scientists’ physicians’ unearned advantages generate greater duties

All citizens have duties to help alleviate avoidable DT not merely proportional to their skills and abilities, but also proportional to their unearned advantages from society. All other things being equal, the greater a citizen’s wealth, intelligence, freedom, health, etc.---the greater her unearned advantages---the greater her responsibility to help alleviate avoidable DT. In particular, because scientists/physicians have received increased, unequal, partially unearned, societal advantages, as compared to many other people, they have increased responsibilities to “give back,” to help promote equal opportunity/protection from harm, as virtually all professional-ethics codes recognize, e.g., [37]. Scientists’/physicians’ increased advantages include government-funded education, research grants, societal protection/licensing for professional careers, and “corners on the market” providing scientific/medical services. These increased advantages arise because of scientists’/physicians’ largely-unearned higher intelligence and resulting higher incomes. Granted, most scientists/physicians have worked hard, but without largely-unearned advantages like high intelligence, good parenting, and educational opportunities, their privileges would have been unachievable. Given such privileges, scientists/physicians and others with such increased advantages have greater responsibilities (than most people) to protect society from harm, especially in areas related to their expertise [35]. To the degree that citizens, including scientists/physicians, do not accept any heightened responsibility---which varies, individual to individual, on the basis of unearned advantages like intelligence, education, etc.---their inaction helps cause avoidable DT, for which they are responsible.

### Fourth argument: DT benefits create DT duties

Citizens, and especially wealthier citizens like scientists/physicians, also bear justice-based responsibilities to help stop DT because, according to the DT-benefits argument, even if epigenetic change is not heritable---all citizens, and scientists/physicians in particular, have justice-based duties to take action on avoidable DT because they benefit from it. How do they benefit? Just as many people have saved money by purchasing sweatshop-produced goods, and just as wealthier people likely purchase more sweatshop goods and therefore have greater responsibility for the harms they cause, something similar holds for DT harms. To see how all citizens, especially wealthier citizens such as scientists/physicians benefit from DT, consider three examples: fossil-fueled automobiles and electricity, pesticide-laden food, and waste incinerators.

### DT harms despite fossil-fuel benefits

At least in Europe and the US, fossil-fueled vehicles cause DT risks because they are responsible for roughly half of all ozone and particulates, neither of which has a safe dose, both of which are especially harmful to children [38,39]. Although asthma is a complex disease with multi-factorial, multi-level origins, particulates alone cause at least $2 billion annually in environmentally-attributable asthma harms to US children, apart from possible developmental decrements. Particulates are at least part of the reason that US pediatric-asthma rates have doubled in the last 10 years [39]. Yet, drivers of fossil-fueled vehicles never compensate their child victims for harms they cause.

Something similar holds for recipients of fossil-fuel-generated electricity. Although coal-fired plants produce roughly 45 percent of US, and 41 percent of global, electricity----and are the largest US source of SO2/mercury/air toxins and major NOX/ozone/particulates sources---virtually all citizens benefit unfairly from this pollution. Why? Coal-generated-electricity users impose developmental risks from pollutants such as mercury, yet they never compensate victims, like US newborns who suffer $9 billion/year in IQ and discounted-lifetime-earnings (DLE) losses from coal-plant-mercury pollution [40]. Instead, fossil-fueled vehicles and electricity plants impose many of their health/economic costs---unpaid by users---on society’s most vulnerable members, children.

### Scientists’/physicians’ greater fossil-fuel benefits generate greater DT duties

Even worse, uncompensated economic benefits that wealthier people---like scientists/physicians---receive from fossil-fueled vehicles and electricity may dwarf benefits to others and explain the greater responsibilities of wealthier people to address DT. Why? Scientists/physicians tend to have higher incomes, and those in the highest-income decile appear to cause about 25 times more fossil-fuel pollutants than those in the lowest-income decile [41]. If so, wealthier people like scientists/physicians may (unintentionally) be responsible for disproportionate injustice from uncompensated DT harms, such as asthma.

In his classic UK-government report, British economist Nicholas Stern [42] estimated that each person in the developed world causes an average of 11 tons/year of carbon-equivalent emissions---roughly $ 935/year in fossil-fuel, climate-only (droughts, floods) effects that kill about 150,000 people/year. Yet global annual outdoor-air-pollution-related deaths, mostly from fossil fuels, are about 1.3 million/year---roughly 10 times greater than climate-related deaths [43]. But if each person in the developed world causes about $900/year climate-related deaths/harms, and if pollution-related fossil-fuel deaths are up to 10 times greater, then developed-world citizen could cause up to (10 x $900) or $9,000/year in fossil-pollution deaths/harms. If so, wealthier people---like physicians/scientists—each might cause up to (25 x $9000) or $225,000 /year in fossil-fuel-pollution damages, including DT [41], mainly to poor people and children, for which they never compensate anyone.

### Greater pesticide and incineration benefits generate greater duties to stop DT

Besides DT caused by fossil-fueled electricity and vehicles, pesticides are widely acknowledged neuro-developmental toxins that constitute a third example of how most people profit unfairly from DT because they buy non-organic food. Although pesticides save consumers money, they impose inequitable DT harms on infants/children, especially farmworker children. Moreover, pesticide-related income losses, alone, are substantial---perhaps $61 billion/year, just from organophosphate-pesticide-induced IQ and DLE losses in children aged 0–5. Once organochlorines and carbamates are included, pesticides harms may rise higher. Why? Data show that for a population of 25.5 million children, aged 0–5, organophosphate pesticides cause losses of about 3,400,000 IQ points/year [44]. Yet the monetary value (DLE) of a one-point IQ loss is about $18,000---if one takes the average of a US EPA estimate of about $11,240 [45]; a Harvard estimate of about $16,500 [46]; a US Centers for Disease Control estimate of about $20,000 [47]; and a Mount Sinai School of Medicine estimate of about $22,300 [40]. Thus $18,000 x 3,400,000 = about $61 billion/year DLE losses caused by organophosphate-exposed young children---if one counts only the 50-percent-highest-exposed children. (This $61 billion/year loss may be an underestimate, given the substantial fraction of overall IQ losses that may be contributed by children with exposures at the lower end of the distribution, and given the relative ubiquity of organophosphate exposures). Although adult food consumers---including scientists/physicians---partly benefit from these losses, they never compensate victims. Yet fairness dictates that consumers pay full costs for their activities/goods, not impose them on innocent children [34].

Incinerator emissions, like lead, constitute another example of how many citizens (unintentionally) gain economic and health benefits from imposing DT risks on children. At least some DT occurs because most citizens (who create garbage) fail to cover full waste-management costs/controls. Because most consumers pay only small household-garbage-pickup fees, the waste is often incinerated in poor neighborhoods---where lead and other emissions impose uncompensated IQ and DLE losses on residents, especially poor/minority children [48].

By benefitting from fossil fuels, pesticide-laden foods, and waste incineration, most citizens are at least partial, unintentional “free riders” who save money and health by imposing their DT risks on poor children [34]. Moreover, because their unfair fossil-fuel, pesticide, incinerator, and other DT-related benefits typically are proportional to their consumption and thus wealth, higher-income groups---like physicians/scientists---achieve greater undeserved gains, and thus bear greater responsibility, for DT-associated harms.

### All major objections fail

In response to previous arguments showing that all citizens---and especially scientists/physicians, because of their greater abilities, training, wealth, etc.---have justice-based duties to help avoid DT, at least 7 questions/objections may arise. These might be called the avoidability, intention, neutrality, economics, excuse, uncertainty, and unfairness objections.

### The avoidability objection

The avoidability question is “how is it possible to determine which DT agents are avoidable?”. This question can be answered only on a case-by-case basis, depending on whether some DT agent is irreplaceable for human needs, e.g., medicines. Moreover, this question’s answer depends in part on the state of science/engineering---including what is known about various chemicals, their DT potential, and possible alternatives to them---and on the ethical theory employed. For instance, all other things being equal, Rawlsian egalitarians would be more likely than Millian utilitarians to say some DT exposure was avoidable, if a much more expensive alternative (to the DT chemical) were available. Why? Rawlsians (more than Millians) require protecting vulnerable minorities, and take less account of the economic costs of protection. Thus, there is no algorithm to answer the avoidability question. As noted earlier, the main (Kantian) point is that “ought implies can.” Therefore, if one cannot avoid some DT exposure, one is not obligated to do so. However, whether an exposure is avoidable requires case-specific, science-specific, ethics-specific assessment.

### The intention objection

What about the intention objection: If I don’t intend to cause harm, how can I be responsible for DT? This objection fails because, as Aristotle [19] noted, people are responsible when their intended acts, culpable ignorance, or inaction causes harm. Why? People are responsible for what should know and who they allow themselves to become. Asking why people failed to stop Hitler (just as we might ask why people have failed to stop avoidable DT, when many people at the time denied Hitler’s atrocities, just as many today deny DT harms), Karl Jaspers [49] and Jean-Paul Sartre [50] charged humankind with “metaphysical guilt.” For what? For not creating themselves, through their attitudes/choices/acts, as the sorts of people who would be help avoid societal harms. To the degree that people’s own inaction/insensitivity has allowed them to become passive, compassionless, or weak---to live in “bad faith”---they are culpable for inaction in the face of great harms, whether Hitler or DT. Hence, to varying degrees (proportional to individual ability, training, profession, income, etc., as argued), everyone has duties to help prevent avoidable DT [49].

### The neutrality objection

Scientists/physicians, however, might have a neutrality, objection. If scientists are supposed to be neutral, value-free, and objective, how can they become activists against DT?

The neutrality objection fails on several logical grounds. First, it relies on the false assumption that objectivity is neutrality; it is not. Objectivity is even-handedness, lack of bias. Otherwise, scientists would have to remain neutral about whether the earth is flat, whether evolution is a fact, or whether anthropogenic climate change exists. Second, if scientists remained neutral, instead of speaking out against DT, they also would err through inconsistency. Why? No good scientists are neutral about poor science. Instead, they use rational debate to help resolve scientific controversies. If so, consistency demands scientists’ using rational debate to assess possible advocacy in areas related to their scientific expertise. Besides, even in doing science, scientists are forced to make hundreds of value-laden, methodological judgments, about everything from proper sample sizes, measurement errors, and possible model biases, to data interpretations [34]. If they did not make methodological value judgments, they could not do science.

Scientists/physicians also must make ethical value judgments---for instance, about allowing avoidable DT---or they violate principles of virtually all codes of professional (scientific) ethics. These codes bind scientists to be unbiased/objective and to promote human welfare/protection. Thus, the American Association for the Advancement of Science (AAAS) emphasizes the “added responsibility of members of the scientific community, individually and through their formal organizations, to speak out” whenever public health or safety is at risk [34,36,37,51]. Besides, if physicians/scientists do not speak out and instead remain neutral, they cannot fulfill their duties of citizenship, duties to help ensure others’ equal protection. Yet no one ought avoid the duties of citizenship. Physicians/scientists therefore do not cease being citizens, just because they are scientists. Indeed, as argued earlier, virtually all ethics codes agree that physicians/scientists’ heightened abilities/training/education arguably give them increased (not reduced) duties as citizens [36,37]. Besides, if physicians/scientists did not have duties to speak out, especially in areas related to their special expertise, worse harms could occur. As Burke put it: All that is necessary for the triumph of evil is that good people do nothing.

Scientists’/physicians’ neutrality on important issues, like DT, also would generate harmful consequences for science itself. Why? The AAAS notes that roughly 75% of US science is funded by industry, 25% by government; that more than half of US-government-funded science is military; and that for every $100 that environmental-health industries spend on their science, government spends about $1 [52]. Consequently, the medical-scientific “playing field” is not level, but often politicized. Declining government science/education funding, and increased special-interest funding, further tilt this playing field, as illustrated by fossil-fuel-industry-funded “science” challenging anthropogenic climate change. The result? Half of Americans believe scientists disagree about whether anthropogenic climate change exists; yet, at least since 1993, no climate scientists publishing in basic-research journals have challenged climate change [53]. Such special-interest “science” helps explains why, even in top medical journals, pharmaceutical-industry-funded studies rarely attribute harmful effects to their drugs, while independent researchers often do so [54], and why chemical-industry-funded studies rarely attribute health damage to their pollutants, while independent researchers often do so [27,55]. Private-interest-science influence also helps explain why university scientists, like Herbert Needleman, were harassed by the lead/gas industry, why the smelting industry harassed Mary Amdur, why the fossil-fuel industry harassed Mike Mann, why the asbestos industry harassed Irving Selikoff, why the beryllium industry harassed Adam Finkel, etc. [34].

Special-interest science also explains why at least 50 percent of environmental epidemiologists---mostly in universities---report polluter harassment after publishing environmental-health research [28]. Given such tilted scientific playing fields, unbiased physicians/scientists who remain neutral serve the status quo, not objectivity. Neutrality allows biased conclusions to become more dominant, science and scientists to be harmed. Because even-handed inquiry (not neutrality) serves objectivity, unbiased scientists need not fear they lack objectivity, if they take action on DT. The opposite is probably true.

### The economics objection

What about a third, or economics, objection. Would preventing avoidable DT cause economic harm? The obvious response is “for whom?” Polluters or innocent children?

The main ethical/logical problem with the economics objection is its begging the questions whether those who seriously harm health should be out of business, whether they have the rights to profit from causing harm. Making this objection is thus a bit like an accused murderer’s claiming (correctly, as already noted) that prosecuting him is not cost-effective. Yet the key issue is justice, not cost. After all, no reasonable person asks whether enforcing basic human rights is economical.

The economics objection also errs factually. As already noted, many scientists have shown that costs of preventing serious health harms----such as DT---are likely less than those caused by allowing them. For instance, French researchers examined economic benefits of lead-abatement/prevention. They discovered lead abatement/prevention costs were far less than health/social costs of addressing lead exposure through screening, special-education expenditures, crime, suffering, and reduced DLE. Although France’s population is one-fifth that of the US, lead-induced IQ losses cause French children’s DLE losses of about 22 Euros/year or $30 billion/year, and French crime losses of 62 Euros/year or $81 billion/year [56]. Similarly, the health, IQ, DLE, etc. losses of US children’s mercury exposures are up to $8 billion/year [46]. More generally, US costs of environmentally-attributable (caused) children’s lead exposure, asthma, cancer, and neurobehavioral problems are up to $55 billion/year [40]. If lead-pollution costs are analogous to those for other developmental toxins, every $1, spent on lead (or other toxin) controls, causes $17-221 in benefits [57]. If so, the economics objection may have little scientific/factual merit.

### The excuse objection

But suppose scientists/physicians have a fourth, or excuse, objection. Do people who do many good things with their lives---raising children, healing patients, serving the poor, doing important research---have rights not to spend time taking action on DT?

While reasonable, the excuse objection errs in ignoring the fact, already argued, that people who cause DT and benefit from it thereby have justice-based duties to compensate for this injustice. Hence they cannot rationally claim excuses for not taking action, any more than robbers can claim ethical excuses for not compensating their victims. Why not? Justice violations require justice-based restitution [58]. Because taking action on DT is a matter of justice, not charity, it is not optional. If not, scientists/physicians---as citizens---have justice-based duties of democratic responsibility, especially if their failure to be politically active in their areas of expertise contributes to DT. Of course, different people’s duties to help address DT differ, depending on their income, health, abilities, etc. Nevertheless, no excuses completely exonerate offenders from justice-based duties to rectify injustice. Otherwise, justice would not exist. Also, it is unreasonable, practically speaking, to offer excuses for completely relinquishing justice-based duties to take action. If such excuses applied to all duties, they would generate question-begging, self-fulfilling prophecies that caused great harm [34].

### The uncertainty objection

Still other thinkers might make a fifth, or uncertainty, objection. How can physicians/scientists take action on DT when the science remains uncertain?

Although reasonable people can disagree about degrees of scientific uncertainty concerning DT, the uncertainty objection is invalid because it commits a logical fallacy, the appeal to ignorance. That is, it assumes that because something has not been proved harmful, it is harmless---that absence of evidence (e.g., for DT) is evidence of the absence (of DT). This objection also relies on at least three faulty assumptions. One is that doing nothing is the way to remain neutral/objective in science---a false claim, already rejected, in response to the neutrality argument. Another erroneous assumption is that the best evidentiary rule is to consider a known toxin innocent of health harm until proved guilty. However, this assumption has been widely challenged, partly through the precautionary principle, because waiting for certainty about a toxin’s harm could cause massive death/disease. Consequently, to protect public health, arguably society should use a “preponderance of evidence rule,” not a “beyond a-reasonable-doubt rule” to assess DT and possible action to prevent harm [27,28]. That is, the objection’s third flawed presupposition is that the best health-science default rule, given scientific uncertainty, is the pure-science rule to minimize false positives. However, many physicians/scientists have shown that because welfare-affecting science must prevent serious harm/injustice, its default rule should be to minimize false negatives. After all, given uncertainty about fire/flood, one does not do nothing, but buys insurance. Given uncertainty about rain, one carries an umbrella. One gets medical check ups, balances her checkbook, exercises, visits the auto mechanic---despite uncertainty about harm. Analogous precautionary requirements also hold for uncertainty about more serious threats, like DT [34].

### The unfairness objection

Even if scientists/physicians admit the importance of taking action, they may have a sixth, or unfairness objection. Is it fair for those----who already have overwhelming research/healing/teaching duties---to have special duties to tackle DT?

Contrary to the unfairness objection, scientists/physicians have such special duties, as argued earlier, because of their special abilities, unearned advantages, greater wealth/consumption, etc. They also have knowledge that few others have and, as Bacon put it, knowledge is power. Those with more power have more responsibility. However, those who make the unfairness argument disagree, likely because of their false assumptions. One false assumption underlying the unfairness objection is that scientists/physicians have “earned” their privileges, hence should have no enhanced duties because of them. However, as already argued, scientists/physicians are not fully “self made.” Because their high IQs (thus better income/education/training/research funding, etc.) are partly unearned/undeserved, matters of chance---they have no right to full benefits from them. If not, as professional codes of ethics point out, scientists have special duties to “give back” to society [7,51], as through tackling DT. But if people ought not profit more than others, from largely unearned/undeserved benefits like high IQs, then fairness dictates scientists/physicians have greater duties to take action than many other people. Noblesse oblige*.* As the scientific research society, Sigma Xi, said: “Because the pathways that we pursue as research scientists are infinite and unfrequented, we cannot police them as we protect our streets and personal property. We depend on those other travelers…along such lonely byways of knowledge” to protect us [51].

Another false assumption often made in the unfairness argument is that, if others are doing little to tackle DT, fairness dictates that I also have little/no responsibility. However, while one’s duties to help avoid societal harm may decrease if others fail to do their fair share, obviously others’ behavior does not dictate ethics. Murder does not become morally acceptable, just because many people murder. To assume others’ action/inaction justifies one’s own action/inaction is to commit the logical fallacy of appeal to the people. Instead, only logic/facts/principles determine what is justified. After all, centuries of racism/sexism have not make them right.

### Scientists/physicians can take action

Citizens/physicians/scientists, however, have no duties to help protect others, in their areas of expertise, unless they are able to do so. “Ought implies can,” as Kant [58,59] put it. How can scientists/physicians take action on DT?

At the group level, organizations like the Society of Toxicology or the American Public Health Association could issue recommendations for DT research/regulatory action. After all, a decade ago, the American Geophysical Union and the American Meteorological Society said we have “collective responsibility” to take action on climate change because we caused it [60]. An analogous claim holds for DT, as already argued.

Working with nongovernmental health/environmental organizations, both local and national---like the Environmental Working Group, or Physicians for Social Responsibility, both of which address children’s health---physicians/scientists also can volunteer their time/expertise. The benefit of such group (and especially local) volunteering is that together, professionals can accomplish what no one, acting alone, could achieve. Recently, for example, the author and other university scientists failed to convince government not to permit a dangerous coal-gasification facility in our already-heavily-polluted area. However, once we gave our information to local doctors, the entire local medical association, hundreds of physicians, voted against the facility. Government was forced to do the same.

Individual physician/scientist action also is useful, such as writing popular essays/blogs on important health topics, whenever they publish analogous professional-journal articles. By “translating” their research for laypeople, scientists/physicians can help promote action on DT. They also can list themselves as media-friendly experts---through their universities, employers, and professional associations---so TV/radio/newspaper/internet reporters easily can contact/interview them. They can serve pro-bono, on local/state/federal advisory boards, such as that for the county health association, the local Sierra Club, or for national groups such as the US EPA’s Science Advisory Board, or committees/boards of the US National Academy of Sciences (NAS). They can give pro-bono testimony in DT court cases. To promote consumer outreach/education on DT, they can speak at school-parent-teacher-association meetings, publish local op-eds, and advise citizens’ groups. School-parent-teacher outlets are especially important because, regardless of people’s politics, as parents they usually are deeply concerned about their children, hence likely to help take action on DT. Moreover, the effort needed for DT action is not great. If historians are correct, only about 14 percent of the early colonists supported the US revolution against England, partly because revolutions are not good for banks and businesses. Imagine the DT-awareness revolution that could occur if 14 percent of parents were mobilized.

At the university or medical-school level, scientists/physicians can encourage administrators, staff, and student organizations to work on phasing out pesticide-laden food, BPA-containing bottles, etc. from university facilities. Scientists/physicians in education can encourage students to do real-world environmental science/health projects, either for course credit or instead of exams. They can teach courses/parts of courses in which students learn to respond to draft health/safety regulations, environmental-impact assessments, and risk assessments----thousands of which are released annually by the US government. In an annual biology course, the author’s students typically receive up to four benefits from their pro-bono projects, responding to draft scientific/government/regulatory analyses. First, students often obtain publications from their work. Second, the victims/impacted communities receive free scientific assistance that helps protect and empowers them, although they cannot pay for help. Third, attorneys often can use this pro-bono student work to assist poor/minority communities harmed by pollution. Fourth, and most important, students who do this scientific work become “vaccinated by social justice,” inspired. Once they realize they can make a difference, they often dedicate at least part of their lives to such pro-bono work, “liberation science,” that helps protect vulnerable people [34].

### Policy suggestions

In the policy arena, how might society take action on DT? One option would be banning all chemicals thought to be developmental toxins until they are proved reasonably safe. However, this strategy appears impractical. Funders of special-interest science probably would block such bans by using tactics like lengthy court challenges. After all, they have blocked most testing/regulation under the 1976 US Toxic Substances Control Act (TSCA). Despite massive growth in chemical production/use, since 1976, TSCA has required testing of only about 200---and has regulated only 5---of roughly 80,000 industrial-agricultural chemicals. Such failures suggest a more moderate strategy is needed.

Another option would be for government to request NAS to study DT and make regulatory/policy recommendations. Like the classic 1993 NAS report on pesticides in the diets of infants and children---which triggered passage of the 1996 US Food Quality Protection Act---a DT study might be the catalyst for protective legislation. The academy could assess DT triggers, criteria, and health consequences. It also could suggest default environmental-health recommendations, until the relevant science is clear---addressing product labeling, temporary exposure-limit safety factors/limits, DT research, testing, compliance assessment, etc. Although admittedly NAS reports take time, whereas taking action is urgent, relying on NAS expertise seems the best long-term strategy, given its credibility.

## Conclusion

While important, deferring to NAS is not sufficient for taking action on DT. Individual citizens/scientists/physicians also must help. To motivate such help, this commentary has offered four main arguments. These focus on citizen duties to act, based on ethical theory, political theory, citizens’ causing DT, and citizens’ benefitting from it. Because of their special abilities/training/wealth/etc., scientists/professionals have heightened responsibilities, as both citizens and professionals, to protect against DT. After all, professionals have many unearned IQ/genetic/upbringing/income privileges from “the natural lottery of life” that have given them unintended but unfair advantages over others [31]. To help compensate for these unearned advantages, physicians/scientists can help level the scientific playing field and take action on DT. As Ghandhi put it, “Whenever I live in a situation where others are in need…whether or not I am responsible for it, I have become a thief” [61]. Like the Brazilian child, DT-threatened children are in need. We are partly culpable. We can be the light that helps banish the darkness around them.

## Abbreviations

AAAS: American Association for the Advancement of Science; HPV: high-production-volume chemicals; EPA: US Environmental Protection Agency; NAS: US National Academy of Sciences; TSCA: US Toxic Substances Control Act.

## Competing interests

The author declares she has no competing financial interests.

## Author’s contributions

The author drafted the first version of the manuscript, and the author revised the manuscript, based on reviewer’s comments. The author read and approved the final manuscript.

## References

[B1] Barouki R Gluckman PD Grandjean P Hanson M Heindel JJ Developmental origins of non-communicable disease: Implications for research and public health Environ Heal 2012 11 42 10.1186/1476-069X-11-42 PMC3384466 22715989

[B2] Skinner MK Manikkam M Guerrero-Bosagna C Epigenetic transgenerational actions of endocrine disruptors Reprod Toxicol 2011 31 337 343 10.1016/j.reprotox.2010.10.012 21055462 PMC3068236

[B3] United Nations Office on Drugs and Crime Action against Transnational Organized Crime and Illicit Trafficking 2011 UN, Geneva

[B4] Shimazono Y The state of the international organ trade Bulletin of the World Health Organization 2007 85 901 980 10.2471/BLT.07.001207 PMC2636295 18278256

[B5] Grandjean P We Have Only One Chance to Develop a Brain 2012 Biomedical Research Park, Barcelona

[B6] Boffetta P McLaughlin JK LaVecchia C Tarone RE Lipworth L Blot WJ False-positive results in cancer epidemiology: a plea for epistemological modesty J Natl Cancer Inst 2008 100 988 995 10.1093/jnci/djn191 18612135 PMC2467434

[B7] Blair A Saracci R Vineis P Cocco P Forastiere F Grandjean P Kogevinas M Kriebel D McMichael A Pearce N Porta M Samet J Sandler DP Costantini AS Vainio H Epidemiology, public health, and the rhetoric of false positives Environ Health Perspect 2009 117 1809 1813 20049197 10.1289/ehp.0901194 PMC2799452

[B8] Grandjean P Eriksen ML Ellegaard O Wallin JA The Matthew effect in environmental-science publications Environ Health 2011 10 96 10.1186/1476-069X-10-96 22074398 PMC3229577

[B9] Landrigan PJ Testimony before the Committee on Environment and Public Works, US Senate 2001 Adelphi University, Garden City, NY

[B10] Schierow LJ The Toxic Substances Control Act, 7–5700 2011 Congressional Research Service, Washington, DC

[B11] Hill AB The environment and disease Proc R Soc Med 1965 58 295 3000 14283879 10.1177/003591576505800503 PMC1898525

[B12] Shrader-Frechette K Randomization and rules for causal inferences in biology Biological Theory 2012 6 1 8 10.1007/s13752-012-0021-y

[B13] Ross WD The Right and the Good. Reprinted with an introduction by Philip Stratton 2002 Oxford University, Oxford

[B14] Kagan S The Limits of Morality 1989 Clarendon Press, Oxford 17n

[B15] Bernal AJ Jirtle RL Epigenomic disruption Birth Defects Res A Clin Mol Teratol 2010 88 938 944 10.1002/bdra.20685 20568270 PMC2945443

[B16] Gluckman PD Hanson MA Low FM The role of developmental plasticity and epigenetics in human health Birth Defects Res C Embryo Today 2011 93 12 18 10.1002/bdrc.20198 21425438

[B17] Grandjean P Landrigan PJ Developmental neurotoxicity of industrial chemicals Lancet 2006 368 2167 2178 10.1016/S0140-6736(06)69665-7 17174709

[B18] Schug TT Janesick A Blumberg B Heindel JJ Endocrine-disrupting chemicals and disease susceptibility J of Steroid Biochemistry and Molecular Biology 2011 127 204 215 10.1016/j.jsbmb.2011.08.007 PMC3220783 21899826

[B19] Aristotle N Ethics, editor and translator, D Ross 1925 Oxford University Press, New York

[B20] Aquinas T On Law, Morality, and Politics, second edition, translator Richard Regan 2002 Hackett, Indianapolis

[B21] Sunstein C Risk and Reason 2002 University Press, Cambridge

[B22] Morgenstern RD Pizer WA Shih JS Jobs versus the environment J Environ Econ Manag 2002 43 412 436 10.1006/jeem.2001.1191

[B23] Dwoskin E Drajem M Regulations create jobs too Bloomberg Businessweek 2012

[B24] Kaplan GA Pamuk ER Lynch JW Cohen RD Balfour JL Inequality and income and mortality in the United States British Medical Journal 1996 312 999 1003 10.1136/bmj.312.7037.999 8616393 PMC2350835

[B25] Trasande L Landrigan PJ Schechter C Public health and economic consequences of methyl mercury toxicity to the developing brain Environ Health Perspect 2005 113 5990 96 10.1289/ehp.7743 PMC1257552 15866768

[B26] Beder S Global Spin 2002 Green Books, Glasgow

[B27] Michaels D Doubt Is Their Product 2008 Oxford University Press, New York

[B28] Wagner W McGarity T Bending Science 2008 Harvard University, Cambridge 158

[B29] Hahnel R Sheeran KA Misinterpreting the Coase Theorem Journal of Economic Issues 2009 43 215 238 10.2753/JEI0021-3624430110

[B30] Mill JS On Liberty and Other Essays 2008 University Press, Oxford

[B31] Rawls J A Theory of Justice 1971 Harvard University Press, Cambridge

[B32] John L Two Treatises of Government 1960 University, Cambridge

[B33] Shrader-Frechette K Gene patents and Lockean constraints Public Affairs Quarterly 2006 20 135 61 16832963

[B34] Shrader-Frechette KS Taking Action, Saving Lives 2007 Oxford University, New York esp. pp. 129; 3–38, 113–149; 159–165; 156; 39–112; 159–165; 164–68; 182–210

[B35] American Association for the Advancement of Science Principles of Scientific Freedom and Responsibility 1980 AAAS, Washington, DC

[B36] American Medical Association Declaration of Professional Responsibility 2001 AMA, San Francisco http://www.ama-assn.org/resources/doc/ethics/decofprofessional.pdf

[B37] Shrader-Frechette KS Ethics of Scientific Research 1994 Rowman and Littlefield, Savage, MD

[B38] European Environment Agency Air Quality and Ancillary Benefits of Climate-Change Policies 2006 EEA, Copenhagen

[B39] Wahlin P Palmgren F Source Apportionment of Particles and Particulates (PM10) Measured by DMA and TROM in a Copenhagen Street Canyon 2000 National Environmental Research Institute, Roskilde, Denmark

[B40] Landrigan PJ Schechter CB Lipton JM Fahs MC Schwartz J Environmental pollutants and disease in American children Environ Health Perspect 2002 110 721 728 10.1289/ehp.02110721 12117650 PMC1240919

[B41] Rabinowitz D Climate injustice. Environmental Justice 2012 5 38 46

[B42] Stern N The Economics of Climate Change 2006 HM Treasury, London

[B43] World Health Association Air Quality and Health 2011 WHO, Copenhagen

[B44] Bellinger DC A strategy for comparing the contributions of environmental chemicals and other risk factors to neurodevelopment of children Environ Health Perspect 2011 120 501 507 10.1289/ehp.1104170 22182676 PMC3339460

[B45] US Environmental Protection Agency Technical Support Document: revision of December 2000 Regulatory Finding on the Emissions of Hazardous Air Pollutants from Electricity Steam Generating Units and the Removal of Coal and Oil-Fired Electric Utility Steam Generating Units from the Section 112(c) List: Reconsideration. October 21 2005 US EPA, Washington, DC

[B46] Rice G Hammitt JK Economic Valuation of Human Health Effects of Controlling Mercury Emissions from US Coal-Fired Power Plants, Northeast States for Coordinated Air Use Management 2005 Harvard Center for Risk Analysis, Cambridge

[B47] Grosse SD How much is an IQ point worth? AERE Newsletter 2007 2007 27 17 21

[B48] World Health Organization Childhood Lead Poisoning 2010 WHO, Geneva

[B49] Jaspers K Ashton EB The Question of German Guilt 1961 Capricorn, New York

[B50] Sartre J-P What Is Literature? trans. Frechtman B 1950 Methuen, London

[B51] Jackson CI Honor in Science 1986 Sigma Xi, New Haven 33

[B52] Koizumi K R&D Trends and Special Analyses, AAAS Reports XXIX, XXVII 2004 American Association for the Advancement of Science’ 2005, Washington, DC

[B53] Oreskes N The scientific consensus on climate change Science 2004 306 1686 10.1126/science.1103618 15576594

[B54] Krimsky S Science in the Private Interest 2004 Rowman and Littlefield, Lanham MD

[B55] Oreskes N Conway E Merchants of Doubt 2010 Bloomsbury, New York 10.1038/465686a 20535183

[B56] Pichery C Bellanger M Zmirou-Navier D Glorennec P Hartemann P Grandjean P Childhood lead exposure in France Environ Health 2011 10 44 10.1186/1476-069X-10-44 21599937 PMC3123267

[B57] Gould E Childhood lead poisoning Environ Health Perspect 2009 117 1162 1167 http://dx.doi.org/10.1289/ehp.0800408 19654928 10.1289/ehp.0800408 PMC2717145

[B58] Kant I Lectures On Ethics, edited by Schneewind JB, translated by Heath P 2001 University Press, Cambridge

[B59] Kant I Critique of Pure Reason, translated by Smith NK 1965 St. Martin’s, New York

[B60] Leung L Ruby L Mearns F Wilby R Regional climate research Bull Am Meteorol Soc 2003 84 89 95 http://dx.doi.org/10.1175/BAMS-84-1-89 10.1175/BAMS-84-1-89

[B61] Goulet D The Cruel Choice 1971 Athenaeum, New York 133

